# Incidentally Detected Persistent Helicobacter heilmannii Gastritis in a Korean Woman With a Normal Endoscopic Examination: Successful Eradication After Salvage Therapy

**DOI:** 10.7759/cureus.117180

**Published:** 2026-09-29

**Authors:** Makaela G Flanagan, Michael Herman

**Affiliations:** 1 College of Osteopathic Medicine, Lincoln Memorial University, Harrogate, USA; 2 Gastroenterology, Borland Groover, Jacksonville, USA

**Keywords:** epigastric discomfort, eradication therapy, gastric biopsy, gastritis, gastroenterology and endoscopy, helicobacter heilmannii, non-helicobacter pylori-helicobacter, persistent infection, salvage therapy

## Abstract

*Helicobacter heilmannii* is an uncommon, non-*Helicobacter pylori* gastric organism associated with chronic gastritis, peptic ulcer disease, gastric mucosa-associated lymphoid tissue lymphoma, and gastric adenocarcinoma. Because the stomach may appear normal endoscopically, diagnosis often depends on histologic examination. Presented is a 59-year-old Korean woman with intermittent epigastric pain and heartburn, as well as a maternal family history of gastric cancer. Upper endoscopy demonstrated a small hiatal hernia with an otherwise normal-appearing stomach. Antral biopsies showed moderate chronic active gastritis with *H. heilmannii* colonization without metaplasia, dysplasia, or malignancy. The patient was treated with clarithromycin-based triple therapy, with symptomatic improvement. Repeat upper endoscopy with gastric mapping four months later demonstrated persistent *H. heilmannii* infection. She then completed 14 days of optimized bismuth quadruple therapy. An additional follow-up upper endoscopy with gastric mapping four months later showed normal gastric histology without residual *Helicobacter*.

This highlights the potential for *H. heilmannii* infection despite a normal endoscopic appearance, the limitations of symptomatic response as evidence of eradication, and the importance of repeat gastric biopsies after treatment failure.

## Introduction

Since the initial description of curved bacilli in patients with chronic gastritis [1], several non-*Helicobacter pylori Helicobacter* species, including *Helicobacter heilmannii*, have been recognized as causes of gastric disease [2-4]. *H. heilmannii* belongs to a group of non-*H. pylori Helicobacter* species that can colonize the human stomach. These organisms are less frequently identified than *H. pylori* and are often grouped under the terms non-*H. pylori Helicobacter* or *H. heilmannii*-like organisms because routine histology cannot reliably distinguish individual species [2-4]. 

Human infection with *Helicobacter* species has been associated with chronic active gastritis, peptic ulcer disease, gastric mucosa-associated lymphoid tissue lymphoma, and gastric adenocarcinoma [2,5-7]. Many *H. heilmannii* infections are believed to be zoonotic in origin, with dogs and cats serving as possible reservoirs [2,8]. Endoscopic findings may be minimal or absent, making gastric biopsy important when clinical suspicion remains despite a normal mucosal appearance [2,5].

Management of *H. heilmannii* is generally drawn from *H. pylori* treatment recommendations [9,10]. Described is a patient with persistent *H. heilmannii* gastritis following clarithromycin-based triple therapy who achieved histologically confirmed eradication after bismuth quadruple therapy. Because evidence guiding *H. heilmannii* diagnosis and treatment remains limited, additional cases are needed to improve recognition and inform future management strategies.

## Case presentation

A 59-year-old Korean woman presented with approximately one year of intermittent epigastric pain and heartburn. Her symptoms occurred randomly but were frequently worsened by spicy foods. She denied dysphagia, nausea, vomiting, melena, rectal bleeding, decreased appetite, or unintentional weight loss. She was not using aspirin or nonsteroidal anti-inflammatory medications. Her family history was notable for gastric cancer involving two maternal relatives. The patient did not confirm or deny animal exposure through cooking or occupation.

Because of her persistent upper gastrointestinal symptoms and family history of gastric cancer, an upper endoscopy was performed. The examination demonstrated a small hiatal hernia and a regular Z-line. The distal esophagus, stomach, gastric antrum, and examined duodenum were without abnormality. Cold-forceps biopsies were obtained from the antrum and gastric body (Figure 1).

**Figure 1:**
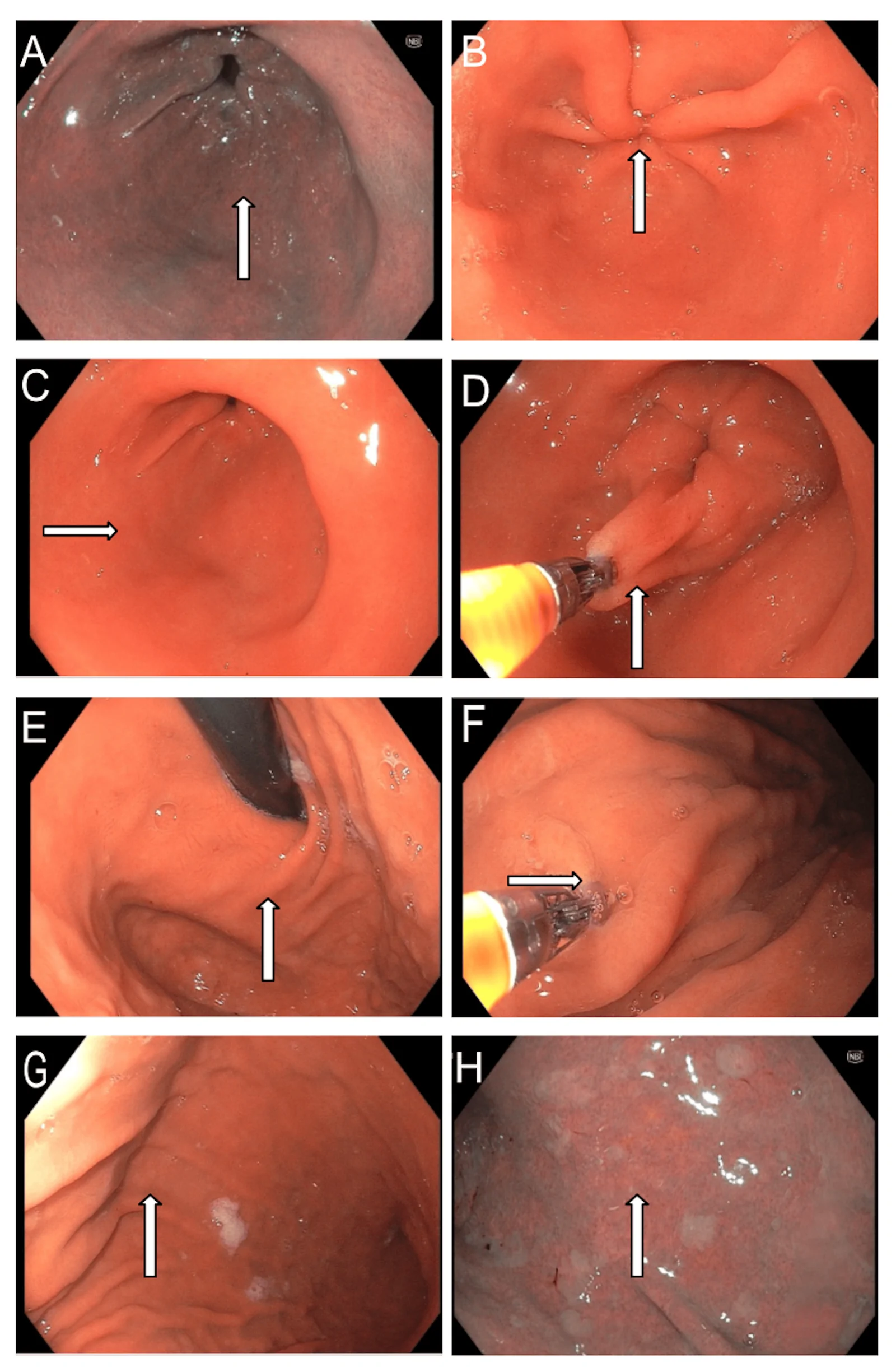
Initial upper endoscopy demonstrating grossly normal upper gastrointestinal mucosa Arrows reference the distal esophagus (A), gastroesophageal junction with a regular Z-line (B), gastric antrum (C), antral biopsy (D), gastric body (E), gastric body biopsy (F), gastric fundus (G), and duodenal bulb (H) appeared endoscopically normal. Cold-forceps biopsies were obtained from the gastric antrum (D) and gastric body (F) for histopathologic evaluation.

Histopathologic examination demonstrated *H. heilmannii*-associated moderate antral gastritis with *H. heilmannii*-like organism colonization (Figure 2). On histological examination, *H. heilmannii*-like organisms can be morphologically larger and thicker than *H. pylori* organisms, with a more distinctive corkscrew shape [5]. No intestinal metaplasia, dysplasia, or malignancy was identified. Duodenal biopsies demonstrated preserved villous architecture without increased intraepithelial lymphocytes or other histopathologic abnormality.

**Figure 2:**
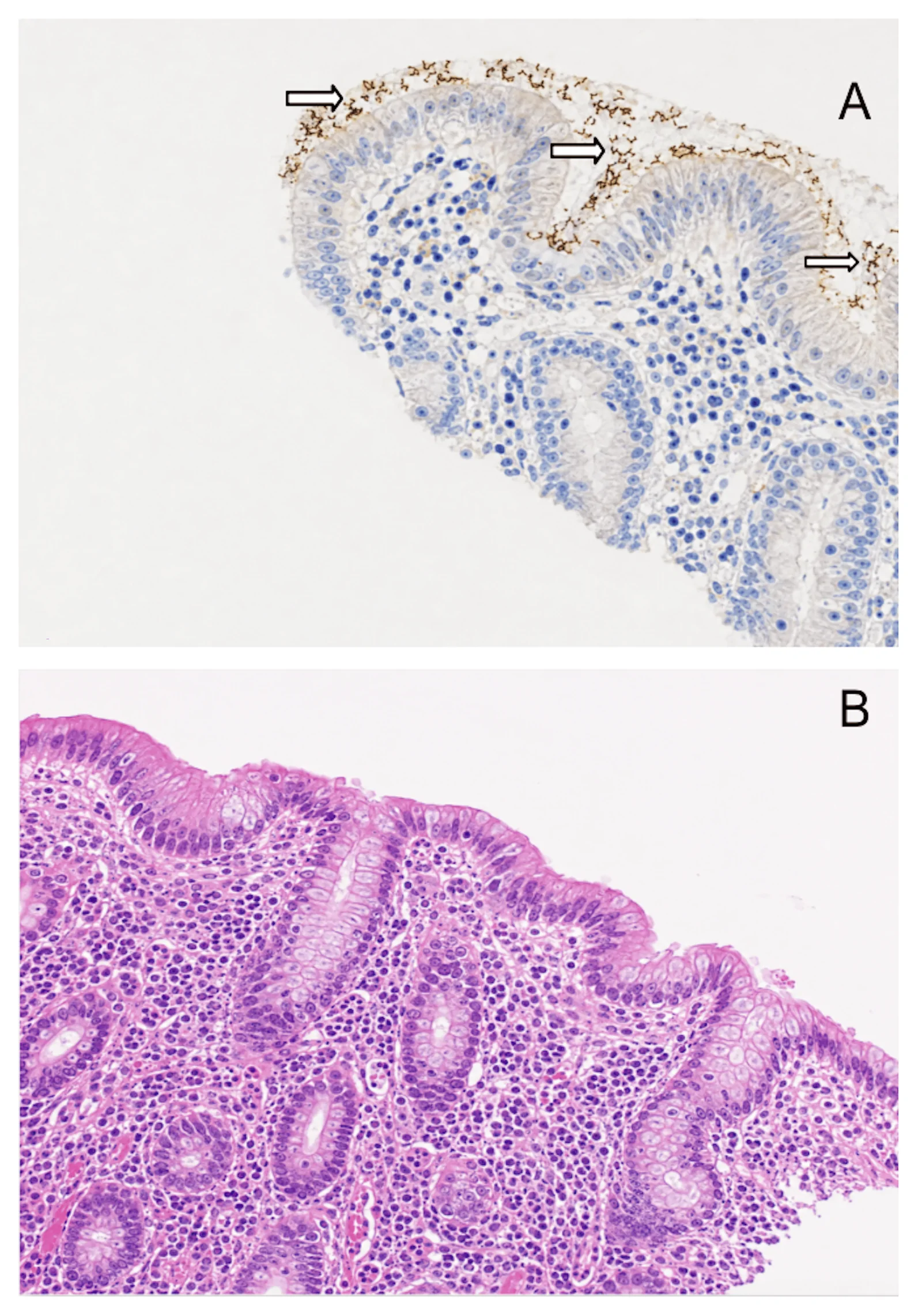
Histopathologic findings of gastric biopsy specimens (A, arrows) *Helicobacter* immunohistochemical stain showing numerous positively stained curved bacillary organisms in brown along the gastric surface epithelium and within the overlying mucus, confirming *Helicobacter*-associated chronic active gastritis. (B) H&E-stained gastric antral mucosa showing chronic active gastritis, with dense lymphoplasmacytic inflammation in the lamina propria, scattered neutrophilic activity involving the foveolar epithelium, and reactive foveolar changes. *Helicobacter* organisms are not readily identifiable on routine H&E staining.

The patient was treated with clarithromycin 500 mg, amoxicillin 1000 mg, and omeprazole 20 mg twice daily for 14 days. This triple therapy was indicated due to the lack of a well-established *H. heilmannii* treatment protocol; therefore, *H. pylori* standard triple therapy was considered the best initial empiric therapy [9]. She reported improvement in her epigastric pain and heartburn after completing treatment.

Due to her family history of gastric cancer and uncertainty regarding noninvasive confirmation of *H. heilmannii* eradication, repeat upper endoscopy was performed four months later. Gastric biopsies were obtained from the antrum, incisura angularis, and gastric body in accordance with a gastric mapping protocol [11,12]. Histopathologic examination continued to demonstrate chronic active gastritis with organisms consistent with *H. heilmannii*, confirming persistent infection despite symptomatic improvement.

She was then treated with 14 days of bismuth quadruple therapy consisting of a proton pump inhibitor twice daily, bismuth subsalicylate four times daily, tetracycline 500 mg four times daily, and metronidazole 500 mg three times daily, as indicated in *H. pylori* treatment [9,10,13].

Another upper endoscopy with gastric mapping was performed four months after completion of bismuth therapy, with the gastric mucosa remaining normal in appearance. Histopathologic examination demonstrated normal gastric mucosa without active gastritis, *Helicobacter* organisms, intestinal metaplasia, dysplasia, or malignancy. These findings confirmed successful eradication of *H. heilmannii*.

## Discussion

This case illustrates several important features of *H. heilmannii* infection. The patient’s stomach appeared entirely normal during the initial upper endoscopy, and the diagnosis was made solely because antral biopsies were obtained. This demonstrates that a normal endoscopic appearance does not exclude clinically relevant *Helicobacter*-associated gastritis [2,5].

The decision to obtain gastric biopsies was particularly important in this patient because she was symptomatic and had a maternal family history of gastric cancer. Although a direct causal relationship between *H. heilmannii* and hereditary gastric cancer risk has not been established, infection with non-*H. pylori Helicobacter* species has been associated with chronic gastritis, gastric lymphoma, and occasionally, gastric adenocarcinoma [2,5-7]. Therefore, identification and eradication of the organism were appropriate [9,10].

The patient experienced symptomatic improvement after clarithromycin-based triple therapy, but repeat histology demonstrated persistent infection. Symptomatic improvement alone should not be interpreted as evidence of eradication [9,10]. Heartburn symptoms may improve due to acid suppression or reduced inflammatory activity while organisms remain present.

The optimal treatment of *H. heilmannii* has not been established through large randomized trials [2]. Most treatment strategies are pulled from established *H. pylori* management [9,10]. Clarithromycin-based triple therapy has historically been used, but prior macrolide exposure and possible clarithromycin resistance due to initial triple therapy may reduce its effectiveness [9,10,13]. After persistence was documented, bismuth quadruple therapy was selected as it avoids reuse of clarithromycin and provides multiple antimicrobial mechanisms [9,10].

Standard *H. pylori* stool antigen and urea breath testing may not reliably detect all non-*H. pylori Helicobacter* species [2]. Alternatively, repeat endoscopy with gastric mapping provided a more definitive method of evaluating infection and confirming eradication [11,12]. The final biopsies demonstrated complete histologic resolution following bismuth therapy.

## Conclusions

*H. heilmannii* gastritis may be present despite a normal-appearing endoscopic examination. Gastric biopsies should be considered in patients with persistent symptoms or increased gastric cancer risk, even if no visible mucosal abnormalities are identified. Clinical improvement after treatment does not confirm organism eradication, and histologic examination of gastric biopsies should be performed. *H. heilmannii* infection should be considered in patients with persistent upper gastrointestinal symptoms despite normal endoscopic appearance.
